# The genome sequence of a drosophilid fruit fly, *Drosophila funebris *(Fabricius, 1789)

**DOI:** 10.12688/wellcomeopenres.20035.1

**Published:** 2023-10-10

**Authors:** Darren J. Obbard, Wellcome Sanger Institute Tree of Life programme, Wellcome Sanger Institute Scientific Operations: DNA Pipelines collective, Tree of Life Core Informatics collective, Darwin Tree of Life Consortium

**Affiliations:** 1 Institute of Ecology and Evolution, The University of Edinburgh, Edinburgh, Scotland, UK

**Keywords:** Drosophila funebris, drosophilid fruit fly, genome sequence, chromosomal, Diptera

## Abstract

We present a genome assembly from an individual male *Drosophila funebris* (drosophilid fruit fly; Arthropoda; Insecta; Diptera; Drosophilidae). The genome sequence is 181.1 megabases in span. Most of the assembly is scaffolded into 7 chromosomal pseudomolecules, including the X and Y sex chromosomes. The mitochondrial genome has also been assembled and is 16.15 kilobases in length.

## Species taxonomy

Eukaryota; Metazoa; Eumetazoa; Bilateria; Protostomia; Ecdysozoa; Panarthropoda; Arthropoda; Mandibulata; Pancrustacea; Hexapoda; Insecta; Dicondylia; Pterygota; Neoptera; Endopterygota; Diptera; Brachycera; Muscomorpha; Eremoneura; Cyclorrhapha; Schizophora; Acalyptratae; Ephydroidea; Drosophilidae; Drosophilinae; Drosophilini; Drosophila; *Drosophila*; *funebris* group; *funebris* subgroup; *Drosophila funebris* (Fabricius, 1789) (NCBI:txid7221).

## Background

**Figure 1.**
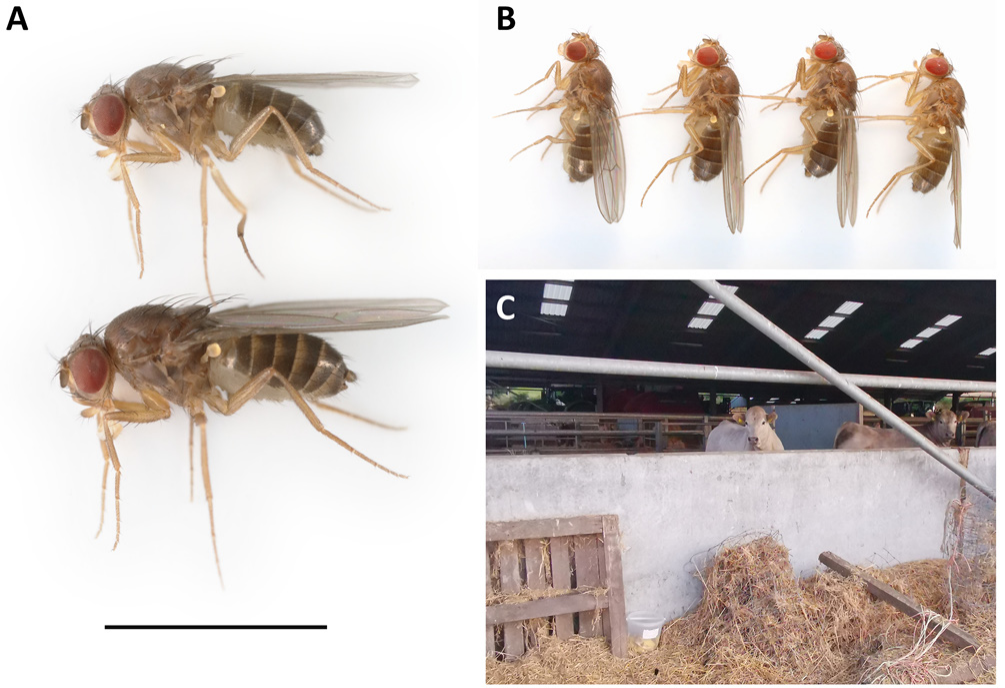
**A:** Male (above) and female (below) *Drosophila funebris* presented with a 3 mm scale bar. **B:** The four lab-reared brothers selected for sequencing. Sample SAMEA12110696 (left) was used for Hi-C sequencing, and sample SAMEA12110696 (second left) was used for PacBio sequencing. **C:** The location for the bait from which the mother of the sequenced flies was collected (Drumness, Perthshire, Scotland; 56.320 N, 3.781 W).

*Drosophila funebris* (Fabricius, 1789) is a medium to large sized (3.5–4.0 mm) mid-brown drosophilid ‘fruit fly’ ( Figure 1A and 1B), and is one of around 30 British and Irish species of *Drosophila* ( Chandler, 2023). In life, it is easily distinguished from the other drosophilid species in the region by its mid-brown colour and relatively long yellowish legs. *Drosophila funebris* (originally placed in *Musca*) was the first drosophilid species to be formally described, and thus provides the type for the genus *Drosophila* and family Drosophilidae. Unfortunately, because it is only distantly related to the laboratory model *D. melanogaster* (which is in the subgenus *Sophophora*), the well-known paraphyly of the genus *Drosophila* cannot be rectified without the undesirable taxonomic instability that would result from renaming *Drosophila melanogaster* to *Sophophora melanogaster* (reviewed in Grimaldi, 2022; O’Grady & Markow, 2009).

Originally from the Palaearctic, *Drosophila funebris* is a now a global – although not generally common – human commensal ( Bächli, 2023; Grimaldi, 2022). Unlike its closest relatives, which are primarily fungus specialists, *D. funebris* is a facultative fungus breeder that is broadly attracted to decaying plant matter, especially that associated with human activity ( Grimaldi, 2022; Shorrocks & Charlesworth, 1980). In the UK, the adults can be seen in any month of the year but are most often recorded in July to November ( GBIF Secretariat, 2023). Specimens have been reared from wild fungi, such as *Boletus edulis* ( Chandler, 2021), but using yeasted fruit baits the adults can be collected across a range of habitats and are often found in the largest numbers in association with farms ( Figure 1C; Basden, 1954; Shorrocks, 1977). In common with many other human-commensal *Drosophila*, *D. funebris* can be maintained in laboratory culture. As a consequence, this species appeared as an early genetic model (e.g. Spencer, 1928; Timofeeff-Rissovsky, 1930) and – like *D. melanogaster* – was studied for variation in chromosomal inversions (e.g. Berrie & Sansome, 1948; Dubinin & Tiniakov, 1946). However, it has only intermittently appeared in experimental studies since, usually with a behavioural or ecological focus ( Arizmendi *et al.*, 2008; Ewing, 1979; Merrell, 1951).

Here we present a chromosomally complete genome sequence for *Drosophila funebris*, derived from the DNA of two male offspring from a wild female collected on yeasted banana bait at Drumness Farm, Perthshire, as part of the Darwin Tree of Life Project. This genome sequence will help to resolve relationships among the Drosophilidae and will further build on the value of this family as a model clade for comparative genomics and molecular evolution. This project is a collaborative effort to sequence all named eukaryotic species in the Atlantic Archipelago of Britain and Ireland.

## Genome sequence report

The genome was sequenced from one male *Drosophila funebris* ( Figure 1) reared at the University of Edinburgh, Scotland, UK (55.92, –3.17). A total of 133-fold coverage in Pacific Biosciences single-molecule HiFi long reads was generated. Primary assembly contigs were scaffolded with chromosome conformation Hi-C data. Manual assembly curation corrected 19 missing joins or mis-joins, reducing the scaffold number by 1.52%.

**Figure 2.**
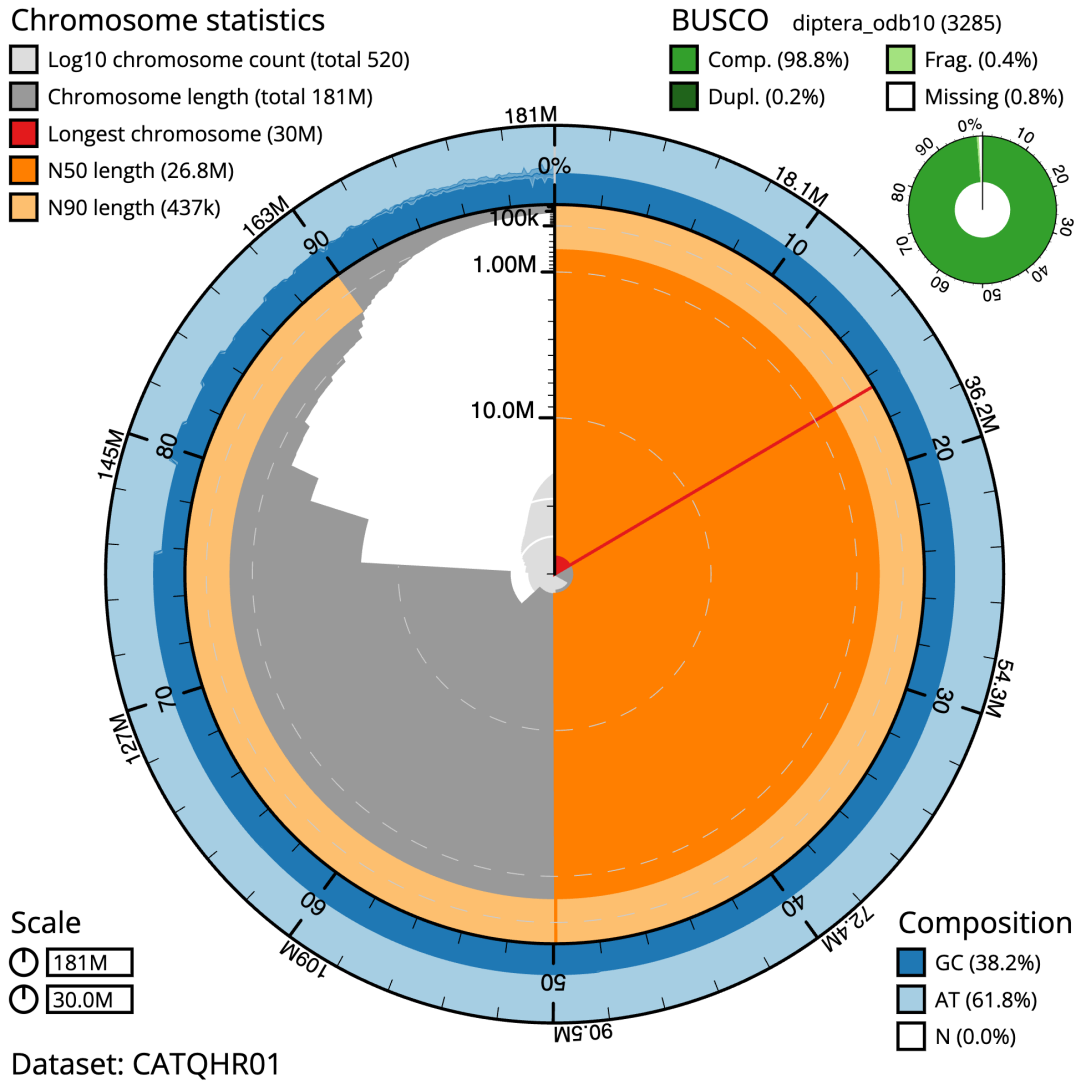
Genome assembly of *Drosophila funebris*, idDroFune2.1: metrics. The BlobToolKit Snailplot shows N50 metrics and BUSCO gene completeness. The main plot is divided into 1,000 size-ordered bins around the circumference with each bin representing 0.1% of the 181,096,236 bp assembly. The distribution of scaffold lengths is shown in dark grey with the plot radius scaled to the longest scaffold present in the assembly (30,028,665 bp, shown in red). Orange and pale-orange arcs show the N50 and N90 scaffold lengths (26,827,462 and 437,266 bp), respectively. The pale grey spiral shows the cumulative scaffold count on a log scale with white scale lines showing successive orders of magnitude. The blue and pale-blue area around the outside of the plot shows the distribution of GC, AT and N percentages in the same bins as the inner plot. A summary of complete, fragmented, duplicated and missing BUSCO genes in the diptera_odb10 set is shown in the top right. An interactive version of this figure is available at https://blobtoolkit.genomehubs.org/view/Drosophila funebris/dataset/CATQHR01/snail.

**Figure 3.**
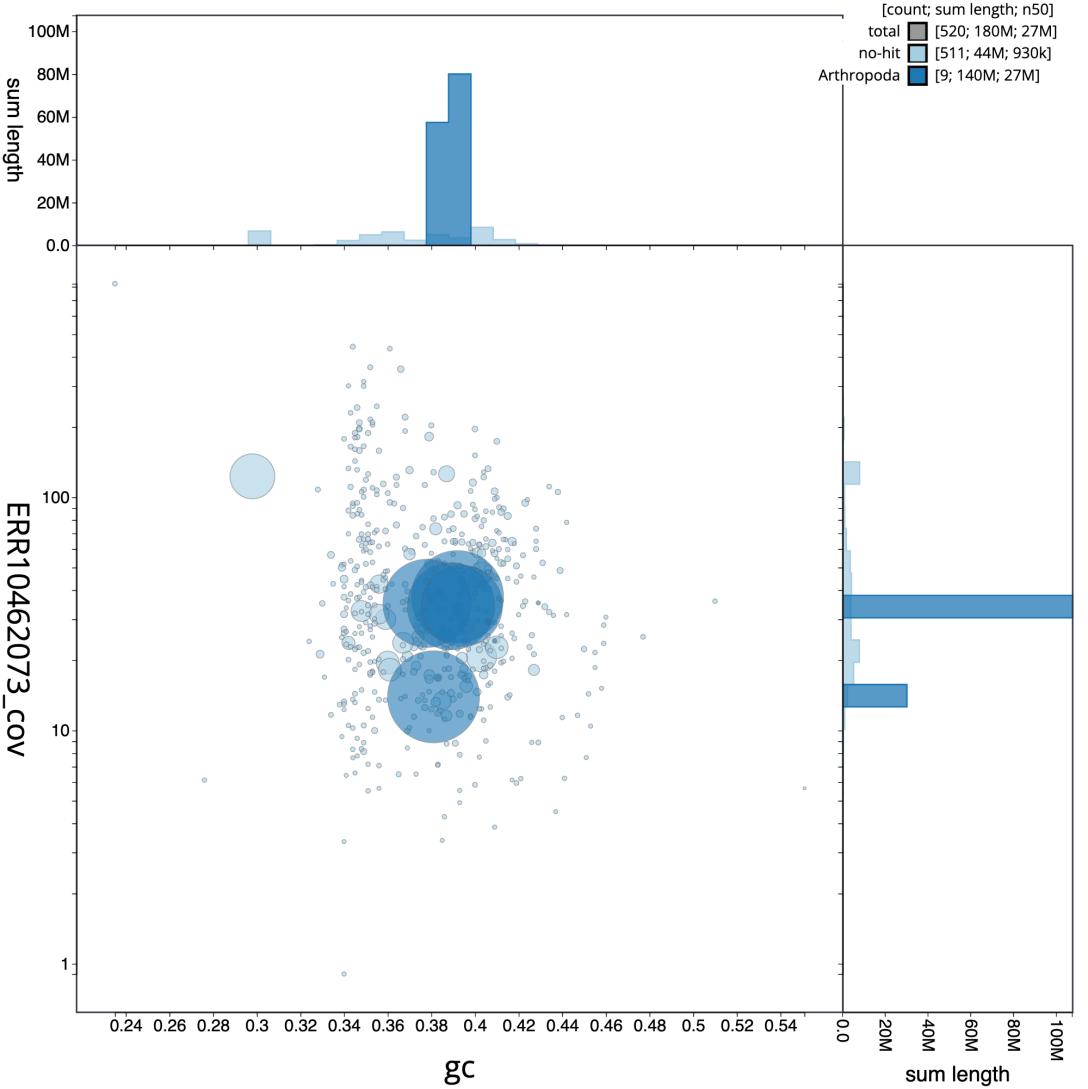
Genome assembly of *Drosophila funebris*, idDroFune2.1: BlobToolKit GC-coverage plot. Scaffolds are coloured by phylum. Circles are sized in proportion to scaffold length. Histograms show the distribution of scaffold length sum along each axis. An interactive version of this figure is available at https://blobtoolkit.genomehubs.org/view/Drosophila funebris/dataset/CATQHR01/blob.

**Figure 4.**
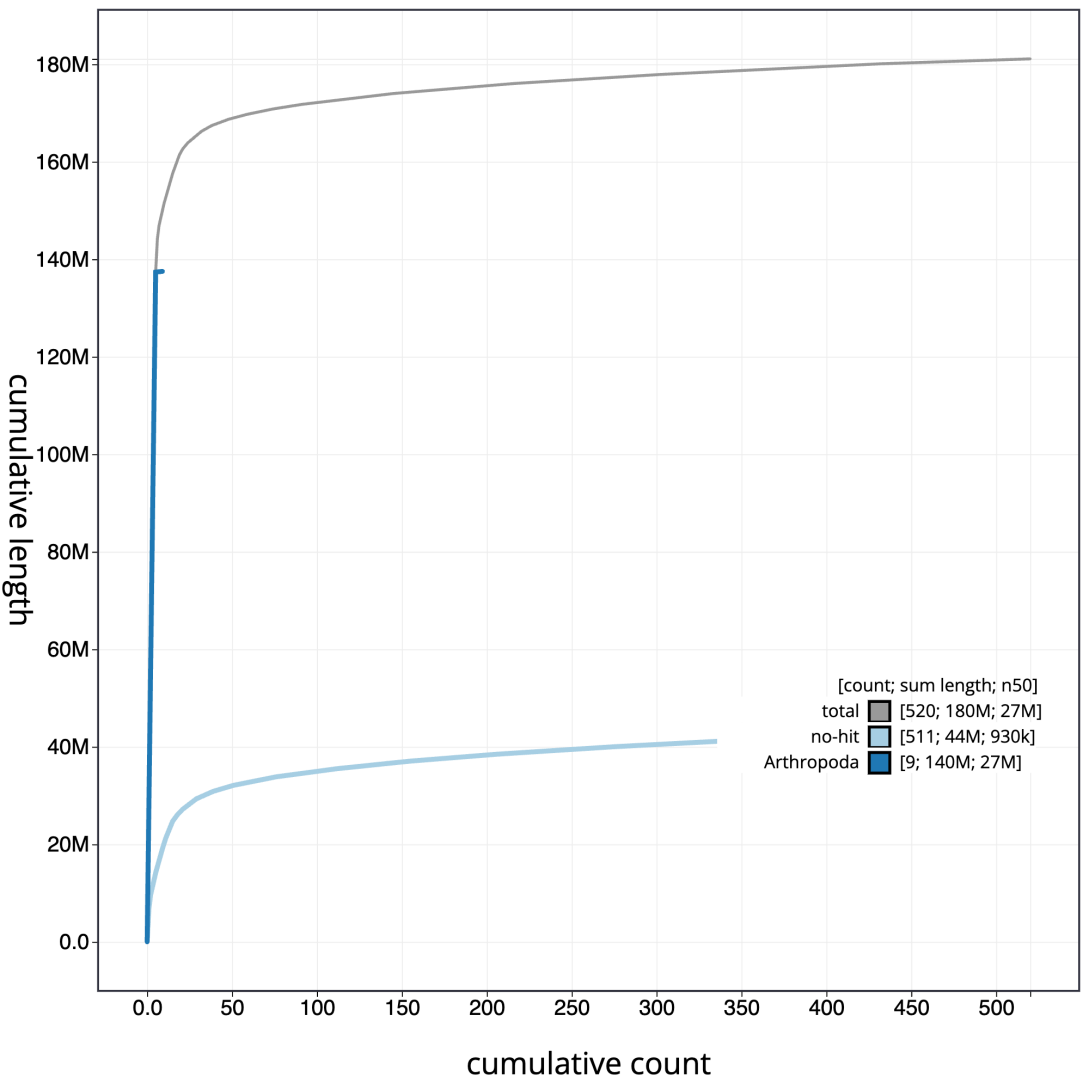
Genome assembly of *Drosophila funebris*, idDroFune2.1: BlobToolKit cumulative sequence plot. The grey line shows cumulative length for all scaffolds. Coloured lines show cumulative lengths of scaffolds assigned to each phylum using the buscogenes taxrule. An interactive version of this figure is available at https://blobtoolkit.genomehubs.org/view/Drosophila funebris/dataset/CATQHR01/cumulative.

**Figure 5.**
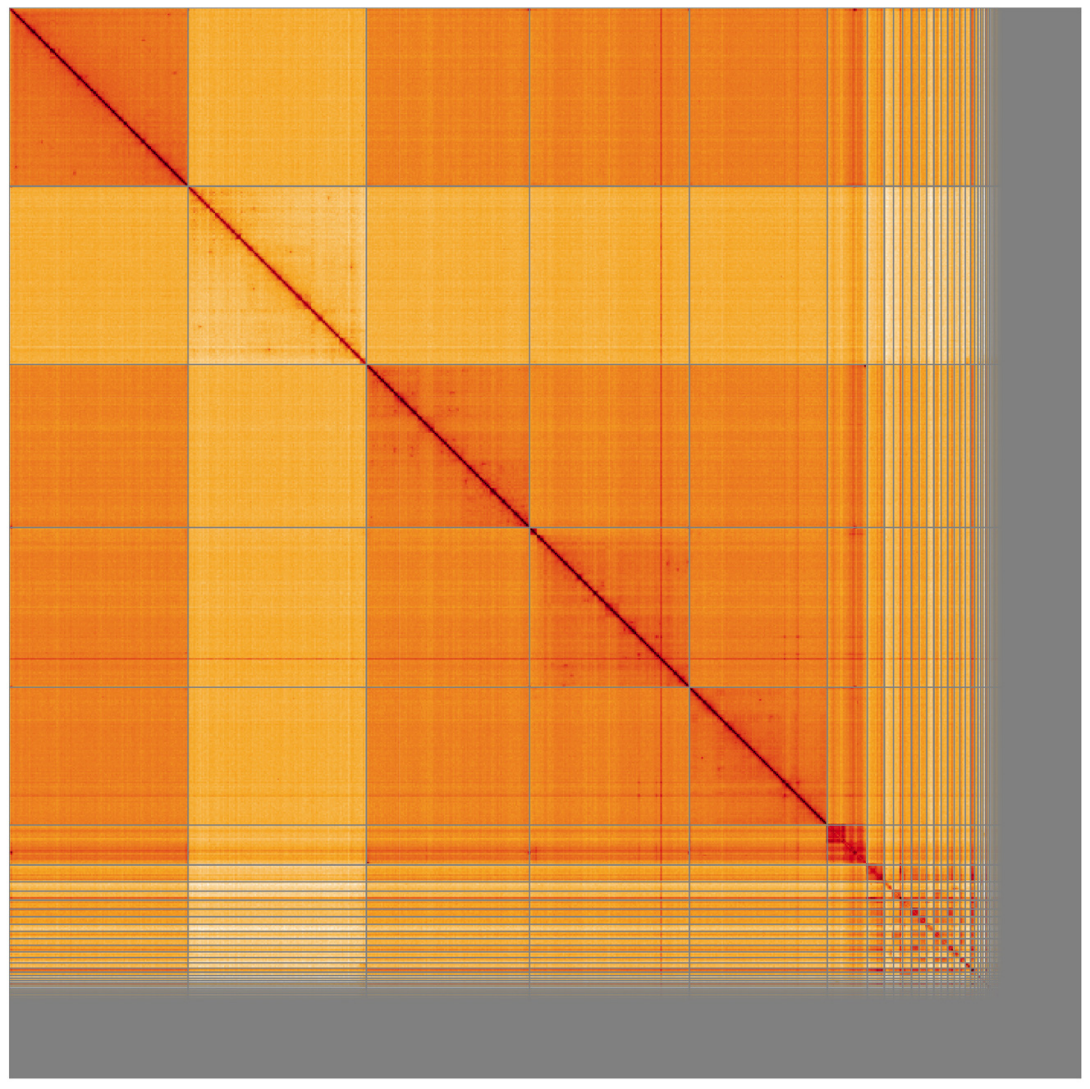
Genome assembly of *Drosophila funebris*, idDroFune2.1: Hi-C contact map of the idDroFune2.1 assembly, visualised using HiGlass. Chromosomes are shown in order of size from left to right and top to bottom. An interactive version of this figure may be viewed at https://genome-note-higlass.tol.sanger.ac.uk/l/?d=dBGzsWLuRGOh668piP9ZlQ.

The final assembly has a total length of 181.1 Mb in 519 sequence scaffolds with a scaffold N50 of 26.8 Mb ( Table 1). A summary of the assembly statistics is shown in Figure 2, while the distribution of assembly scaffolds on GC proportion and coverage is shown in Figure 3. The cumulative assembly plot in Figure 4 shows curves for subsets of scaffolds assigned to different phyla. Most (96.46%) of the assembly sequence was assigned to 7 chromosomal-level scaffolds, representing 5 autosomes and the X and Y sex chromosomes. Chromosome-scale scaffolds confirmed by the Hi-C data are named in order of size ( Figure 5; Table 2). The X and Y chromosomes were identified by read coverage. Repetitive scaffolds of the Y chromosome whose location could not be determined were left as unlocalised sequences. While not fully phased, the assembly deposited is of one haplotype. Contigs corresponding to the second haplotype have also been deposited. The mitochondrial genome was also assembled and can be found as a contig within the multifasta file of the genome submission.

**Table 1.** Genome data for *Drosophila funebris*, idDroFune2.1.

Project accession data		
Assembly identifier	idDroFune2.1	
Species	*Drosophila funebris*	
Specimen	idDroFune2	
NCBI taxonomy ID	7221	
BioProject	PRJEB57268	
BioSample ID	SAMEA12110453	
Isolate information	idDroFune2, male: whole organism (DNA sequencing) idDroFune1, male: whole organism (Hi-C scaffolding)	
Assembly metrics *		*Benchmark*
Consensus quality (QV)	62.9	*≥ 50*
*k*-mer completeness	100%	*≥ 95%*
BUSCO **	C:98.8%[S:98.5%,D:0.2%], F:0.4%,M:0.8%,n:3,285	*C ≥ 95%*
Percentage of assembly mapped to chromosomes	96.46%	*≥ 95%*
Sex chromosomes	X and Y chromosomes	*localised homologous * *pairs*
Organelles	Mitochondrial genome assembled	*complete single alleles*
Raw data accessions		
PacificBiosciences SEQUEL II	ERR10462073	
Hi-C Illumina	ERR10466807	
Genome assembly		
Assembly accession	GCA_958295475.1	
*Accession of alternate haplotype*	GCA_958295065.1	
Span (Mb)	181.1	
Number of contigs	882	
Contig N50 length (Mb)	0.7	
Number of scaffolds	519	
Scaffold N50 length (Mb)	26.8	
Longest scaffold (Mb)	30.03	

* Assembly metric benchmarks are adapted from column VGP-2020 of “ Table 1: Proposed standards and metrics for defining genome assembly quality” from ( Rhie *et al.*, 2021). ** BUSCO scores based on the diptera_odb10 BUSCO set using v5.3.2. C = complete [S = single copy, D = duplicated], F = fragmented, M = missing, n = number of orthologues in comparison. A full set of BUSCO scores is available at https://blobtoolkit.genomehubs.org/view/Drosophila funebris/dataset/CATQHR01/busco.

**Table 2.** Chromosomal pseudomolecules in the genome assembly of *Drosophila funebris*, idDroFune2.

INSDC accession	Chromosome	Length (Mb)	GC%
OY282423.1	1	30.03	39.0
OY282425.1	2	27.41	38.0
OY282426.1	3	26.83	39.0
OY282427.1	4	23.16	39.5
OY282428.1	5	6.71	30.0
OY282424.1	X	29.94	38.0
OY282422.1	Y	2.85	40.5
OY282429.1	MT	0.02	23.5

The estimated Quality Value (QV) of the final assembly is 62.9 with *k*-mer completeness of 100%, and the assembly has a BUSCO v5.3.2 completeness of 98.8% (single = 98.5%, duplicated = 0.2%), using the diptera_odb10 reference set ( *n* = 3,285).

Metadata for specimens, spectral estimates, sequencing runs, contaminants and pre-curation assembly statistics can be found at https://links.tol.sanger.ac.uk/species/7221.

## Methods

### Sample acquisition and nucleic acid extraction

*Drosophila funebris* specimens were first-generation male progeny from a wild-collected female. The sequenced flies were reared on *Drosophila* banana malt medium at the University of Edinburgh (latitude 55.92, longitude –3.17), and were harvested on 2021-10-02. The mother was collected from banana/yeast bait at a farm in Perthshire, Scotland (56.32, –3.78) on 2021-08-05, and had been mated prior to collection. The flies were collected and identified by Darren Obbard (University of Edinburgh), and species identification was confirmed by examination of the progeny. Each living anaesthetised fly was placed directly into the collection tube and frozen from live at –80°C. The sample with specimen ID SAN00001902 (ToLID idDroFune2) was used for DNA sequencing and the sample with specimen ID SAN00001901 (ToLID idDroFune1) was used for Hi-C scaffolding.

DNA was extracted at the Tree of Life laboratory, Wellcome Sanger Institute (WSI). The idDroFune2 sample was weighed and dissected on dry ice. Tissue from the whole organism was disrupted using a Nippi Powermasher fitted with a BioMasher pestle. High molecular weight (HMW) DNA was extracted using the Qiagen MagAttract HMW DNA extraction kit. HMW DNA was sheared into an average fragment size of 12–20 kb in a Megaruptor 3 system with speed setting 30. Sheared DNA was purified by solid-phase reversible immobilisation using AMPure PB beads with a 1.8X ratio of beads to sample to remove the shorter fragments and concentrate the DNA sample. The concentration of the sheared and purified DNA was assessed using a Nanodrop spectrophotometer and Qubit Fluorometer and Qubit dsDNA High Sensitivity Assay kit. Fragment size distribution was evaluated by running the sample on the FemtoPulse system.

### Sequencing

Pacific Biosciences HiFi circular consensus DNA sequencing libraries were constructed according to the manufacturers’ instructions. DNA sequencing was performed by the Scientific Operations core at the WSI on a Pacific Biosciences SEQUEL II (HiFi) instruments. Hi-C data were generated from tissue of idDroFune1 using the Arima2 kit and sequenced on the Illumina NovaSeq 6000 instrument.

### Genome assembly, curation and evaluation

Assembly was carried out with HiCanu ( Nurk *et al.*, 2020) and haplotypic duplication was identified and removed with purge_dups ( Guan *et al.*, 2020). The assembly was then scaffolded with Hi-C data ( Rao *et al.*, 2014) using YaHS ( Zhou *et al.*, 2023). The assembly was checked for contamination and corrected as described previously ( Howe *et al.*, 2021). Manual curation was performed using HiGlass ( Kerpedjiev *et al.*, 2018) and Pretext ( Harry, 2022). The mitochondrial genome was assembled using MitoHiFi ( Uliano-Silva *et al.*, 2023), which runs MitoFinder ( Allio *et al.*, 2020) or MITOS ( Bernt *et al.*, 2013) and uses these annotations to select the final mitochondrial contig and to ensure the general quality of the sequence.

A Hi-C map for the final assembly was produced using bwa-mem2 ( Vasimuddin *et al.*, 2019) in the Cooler file format ( Abdennur & Mirny, 2020). To assess the assembly metrics, the *k*-mer completeness and QV consensus quality values were calculated in Merqury ( Rhie *et al.*, 2020). This work was done using Nextflow ( Di Tommaso *et al.*, 2017) DSL2 pipelines “sanger-tol/readmapping” ( Surana *et al.*, 2023a) and “sanger-tol/genomenote” ( Surana *et al.*, 2023b). The genome was analysed within the BlobToolKit environment ( Challis *et al.*, 2020) and BUSCO scores ( Manni *et al.*, 2021; Simão *et al.*, 2015) were calculated.

Table 3 contains a list of relevant software tool versions and sources.

**Table 3.** Software tools: versions and sources.

Software tool	Version	Source
BlobToolKit	4.1.7	https://github.com/blobtoolkit/blobtoolkit
BUSCO	5.3.2	https://gitlab.com/ezlab/busco
Hicanu	2.2	https://github.com/marbl/canu
HiGlass	1.11.6	https://github.com/higlass/higlass
Merqury	MerquryFK	https://github.com/thegenemyers/MERQURY.FK
MitoHiFi	2	https://github.com/marcelauliano/MitoHiFi
PretextView	0.2	https://github.com/wtsi-hpag/PretextView
purge_dups	1.2.3	https://github.com/dfguan/purge_dups
sanger-tol/genomenote	v1.0	https://github.com/sanger-tol/genomenote
sanger-tol/readmapping	1.1.0	https://github.com/sanger-tol/readmapping/tree/1.1.0
yahs-1.2a.2	yahs-1.2a.2	https://github.com/c-zhou/yahs

### Wellcome Sanger Institute – Legal and Governance

The materials that have contributed to this genome note have been supplied by a Darwin Tree of Life Partner. The submission of materials by a Darwin Tree of Life Partner is subject to the **‘Darwin Tree of Life Project Sampling Code of Practice’**, which can be found in full on the Darwin Tree of Life website here. By agreeing with and signing up to the Sampling Code of Practice, the Darwin Tree of Life Partner agrees they will meet the legal and ethical requirements and standards set out within this document in respect of all samples acquired for, and supplied to, the Darwin Tree of Life Project.

Further, the Wellcome Sanger Institute employs a process whereby due diligence is carried out proportionate to the nature of the materials themselves, and the circumstances under which they have been/are to be collected and provided for use. The purpose of this is to address and mitigate any potential legal and/or ethical implications of receipt and use of the materials as part of the research project, and to ensure that in doing so we align with best practice wherever possible. The overarching areas of consideration are:

•   Ethical review of provenance and sourcing of the material

•   Legality of collection, transfer and use (national and international) 

Each transfer of samples is further undertaken according to a Research Collaboration Agreement or Material Transfer Agreement entered into by the Darwin Tree of Life Partner, Genome Research Limited (operating as the Wellcome Sanger Institute), and in some circumstances other Darwin Tree of Life collaborators.

## Data Availability

European Nucleotide Archive: *Drosophila funebris*. Accession number PRJEB57268; https://identifiers.org/ena.embl/PRJEB57268. ( Wellcome Sanger Institute, 2023) The genome sequence is released openly for reuse. The *Drosophila funebris* genome sequencing initiative is part of the Darwin Tree of Life (DToL) project. All raw sequence data and the assembly have been deposited in INSDC databases. The genome will be annotated using available RNA-Seq data and presented through the Ensembl pipeline at the European Bioinformatics Institute. Raw data and assembly accession identifiers are reported in Table 1.

## Author information

Members of the Wellcome Sanger Institute Tree of Life programme are listed here: https://doi.org/10.5281/zenodo.4783585.

Members of Wellcome Sanger Institute Scientific Operations: DNA Pipelines collective are listed here: https://doi.org/10.5281/zenodo.4790455.

Members of the Tree of Life Core Informatics collective are listed here: https://doi.org/10.5281/zenodo.5013541.

Members of the Darwin Tree of Life Consortium are listed here: https://doi.org/10.5281/zenodo.4783558.
