# Supplementary material for: The genome sequence of a drosophilid fruit fly, Drosophila funebris (Fabricius, 1789)
Source: Wellcome Open Res. 2023 Oct 10;8:437. [Version 1] doi: 10.12688/wellcomeopenres.20035.1 (PMC11126897; doi:10.12688/wellcomeopenres.20035.1)
Supplement: Peer review history

# Reviewer report: Reviewer response for version 1

**DOI:** 10.21956/wellcomeopenres.22190.r70856

**Published:** 2024-05-28

**Authors:** Lucio Navarro-Escalante

**Affiliations:** 1 Department of Entomology, Federacion Nacional de Cafeteros de Colombia, Caldas, Colombia; 2 Department of Molecular Biosciences, The University of Texas at Austin, Austin, Texas, USA

This paper describes a high quality genome assembly of the drosophilid fruit fly *Drosophila funebris* using PacBio HiFi long reads and Hi-C scaffolding. DNA samples for this sequencing were obtained from male specimens. The genome is presented at chromosomal level and contains the X and Y sex chromosomes, as well as the mitochondrial genome as a single contig. Genome assembly completeness was also addressed using BUSCO. Primary PacBio sequencing resulted in a relatively high genome coverage (>100 fold) that is more than enough to assemble really good contigs and possibly avoid the need for polishing with Illumina reads. In general, the  method descriptions are mostly described with enough details and are easy to follow. Genome assembly report is clearly described and offers good details in tables and figures. I really appreciate the effort to report the pre-curated genome assembly statistics. Despite the few comments I have below. 

**Comments:**

 Genome sequence report:

- The total amount of Hi-C Illumina data should be reported in the main text or within tables.
- The sequence coverage data used to discriminate sex chromosomes from autosomal chromosomes should be presented in some way. They could be presented in a figure showing perhaps mean coverage across each chromosome or be reported in table 2. 

Method section:

- A brief description of the steps followed for checking sequence contamination and assembly correction should be included. Authors indicate they followed the methods by Howe et al. (2021), which means they used the gEVAL approach, a non-portable tool that may not allow for detailed replication outside its own infrastructure.
- For all bioinformatic tools used here, authors should report whether the “default” or any particular options were applied in the analysis.

Are sufficient details of methods and materials provided to allow replication by others?

Yes

Is the rationale for creating the dataset(s) clearly described?

Yes

Are the datasets clearly presented in a useable and accessible format?

Yes

Are the protocols appropriate and is the work technically sound?

Yes

Reviewer Expertise:

Insect genome assembly,  gene annotation, insect-microbial symbiosis.

I confirm that I have read this submission and believe that I have an appropriate level of expertise to confirm that it is of an acceptable scientific standard.

## References

[1] : Ensembl 2021. *Nucleic Acids Res* .2021;49(D1) : 10.1093/nar/gkaa942 D884-D891 10.1093/nar/gkaa942 33137190 PMC7778975

# Reviewer report: Reviewer response for version 1

**DOI:** 10.21956/wellcomeopenres.22190.r74681

**Published:** 2024-05-25

**Authors:** Qingsong Zhou

**Affiliations:** 1 Chinese Academy of Sciences, Beijing, China

The authors presented the genome of *Drosophila funebris* (Fabricius, 1789) at the chromosome level and clarified the relationship between *D. funebris* and *D. melanogaster*, providing useful information to understand the significance of this study. The methods and software used in the analysis workflow were effectively employed to generate the reference genome of Diptera. The BlobToolKit, BUSCO assessments, and QV value indicate that the genome is of high quality. It would be better to provide more detailed information for the annotated genes. In conclusion, this work contributes to the reference genome resources of Diptera, especially for the well-known "fruit fly" genus *Drosophila*.

Are sufficient details of methods and materials provided to allow replication by others?

Yes

Is the rationale for creating the dataset(s) clearly described?

Yes

Are the datasets clearly presented in a useable and accessible format?

Yes

Are the protocols appropriate and is the work technically sound?

Yes

Reviewer Expertise:

Entomology, genomics

I confirm that I have read this submission and believe that I have an appropriate level of expertise to confirm that it is of an acceptable scientific standard.

# Reviewer report: Reviewer response for version 1

**DOI:** 10.21956/wellcomeopenres.22190.r70850

**Published:** 2024-01-02

**Authors:** Héloïse Bastide

**Affiliations:** 1 Laboratoire Évolution, Génomes, Comportement et Écologie, CNRS, IRD, Université Paris-Saclay - Institut Diversité, Gif-sur-Yvette, France

This paper by Darren J. Obbard presents a new reference genome for *Drosophila funebris*, the type species of the genus *Drosophila*. The latter has been one of the most extensively studied genus at the molecular level with twelve sequenced genomes available as of 2007. This genome sequence was obtained from a single male. It is complete with 7 chromosome-scale scaffolds including the X and Y chromosomes and the mitochondrial genome is also made available as a single contig.

 Overall, the assembly metrics show a high-quality sequenced genome with a consensus quality value of 62,9, a *k*-mer completeness of 100% and a BUSCO score of 98,8%. 96,46% of the assembly was assigned to the 7 chromosomes. While this new genome is clearly of great interest for the scientific community, it would be useful for the readers to know how it compares to already published genome assemblies of *D. funebris* (Kim et al. 2021 Elife).

 In the background section, the author raises the problem of the paraphyly of the genus *Drosophila* on the nomenclature of *D. melanogaster*. While the reference given is relevant (Markow and O’Grady 2009), more recent studies have explicitly treated this question within the broader context of Drosophilidae relationships (Yassin 2013 Syst. Entomol; O’Grady and DeSalle 2018 Genetics). Finally, the sentence “This genome sequence will help to resolve relationships among the Drosophilidae and will further build on the value of this family as a model clade for comparative genomics and molecular evolution“ sounds a bit mysterious to me as the placement of *D. funebris* within the paraphyletic genus *Drosophila* has long been confirmed even using whole genome sequences (Suvorov et al. 2021 Curr. Biol.). I am thus curious to know how specifically this high-quality assembly will improve (and resolve) relationship inferences among the Drosophilidae.

Are sufficient details of methods and materials provided to allow replication by others?

Yes

Is the rationale for creating the dataset(s) clearly described?

Yes

Are the datasets clearly presented in a useable and accessible format?

Yes

Are the protocols appropriate and is the work technically sound?

Yes

Reviewer Expertise:

Population genetics, evolutionary genomics, insects.

I confirm that I have read this submission and believe that I have an appropriate level of expertise to confirm that it is of an acceptable scientific standard.
